# Feasibility of implementing a preventive physical exercise programme recommended by general practitioners in cardiovascular risk patients: A pre-post comparison study

**DOI:** 10.1080/13814788.2020.1760836

**Published:** 2020-05-22

**Authors:** Francisco Javier López-Román, Francisca I. Tornel-Miñarro, Eloisa Delsors-Merida-Nicolich, Lourdes Fernández-López, María Teresa Martínez-Ros, Esther García Sánchez, Asensio López-Santiago

**Affiliations:** a Family Medicine, Dirección General de Planificación, Investigación, Farmacia y Atención al Ciudadano, Consejería de Salud, Murcia, Spain; b Pharmacy, Dirección General de Planificación, Investigación, Farmacia y Atención al Ciudadano, Consejería de Salud, Murcia, Spain; c Family Medicine, Primary Health Care Center Jesús Marín, Murcia, Spain; d Family Medicin, Dirección General de Planificación, Investigación, Farmacia y Atención al Ciudadano, Consejería de Salud, Murcia, Spain; f Exercise Professionals, Dirección General de Planificación, Investigación, Farmacia y Atención al Ciudadano, Consejería de Salud, Murcia, Spain;; g Family Medicine, Servicio Murciano de Salud, Región de Murcia, Murcia, Spain

**Keywords:** Physical exercise, health promotion programmes, primary care, hypertension, dyslipidaemia

## Abstract

**Background:**

Physical inactivity implies a significant individual and society health burden.

**Objectives:**

To assess the feasibility of implementing a preventive physical exercise (PE) programme for the general population and to analyse changes in fitness-related variables and quality of life.

**Methods:**

Pre-post comparison study in which general practitioners and nurses recommended PE to participants with sedentary behaviour and hypertension or dyslipidaemia attending in primary care for primary prevention of ischaemic cardiovascular disease. Eligible participants were referred to a PE programme (10 weeks, three days a week, a total of 30 sessions of one-hour duration). Data was collected for five years (2013–2017). Outcome measures were body weight, body mass index (BMI), physical condition (aerobic fitness, muscle strength, flexibility, balance), and quality of life (SF-36).

**Results:**

The PE programme was offered to 6,140 eligible subjects; 5,077 (82.7%) accepted to participate and received a recommendation; 3,656 (69.6% women) started the programme and 2,962 subjects (80.9% women) finished the programme. After 10 weeks, there were significant improvements (mean difference, 95% CI) in aerobic fitness (2.55 ml/min/kg, 2.32–2.79), muscle strength (0.62 m, 0.57 to 0.67), flexibility (2.34 cm, 2.06 to 2.63) and balance (−0.46 falls, −0.60 to −0.33) as well as significant decreases in body weight (−0.41 kg, −0.64 to −0.17) and BMI (−0.27 kg/m^2^, −0.34 to −0.20).

**Conclusion:**

Implementation of a government-supported PE programme for the general population recruited in the primary care setting and recommended by healthcare professionals is feasible, and was associated with health benefits, mainly improvements in physical fitness.

KEY MESSAGES

- A community programme of physical exercise, with free participation, recommended by primary healthcare professionals to cardiovascular risk patients was implemented.
- Over five years, 3,656 cardiovascular risk patients participated in the programme.
- After 10 weeks, positive effects on physical fitness and decreases in body weight and body mass index were recorded.

## Introduction

Physical exercise (PE) is an important measure for the prevention of chronic conditions [1–4], particularly to reduce the risk of adverse outcomes in patients with cardiovascular diseases [5–9]. Community-based interventions, about the community as the setting for the intervention, for increasing levels of PE of the population at large, have been a focus of growing interest [10,11]. The effectiveness of community-based PE programmes is hampered by the vast array of approaches, settings, formats and measures reported in the literature [12–16]. For these interventions to be effective, governmental commitment and support at all levels are indispensable. A community-based programme promoting PE has been developed by the government of the Region of Murcia, Spain (an autonomous community in the southeast on the Mediterranean coast, with a population of 1.4 million) [17]. The programme is based on the advice of PE by general practitioners and nurses to patients with cardiovascular risk factors who could benefit from improvement in physical fitness.

We here report the results obtained after implementation of the governmental programme in participants with a sedentary lifestyle and hypertension or dyslipidaemia attended in primary care for primary prevention of their ischaemic cardiovascular disease. The primary objective of the study was to assess the feasibility of implementing a preventive physical exercise (PE) programme offered to eligible subjects from the general population and to analyse changes in the physical condition and quality of life of participants. The secondary objective was to assess the opinion of participants regarding the benefits derived from the programme.

## Method

### Study design and setting

A pre-post comparison study was designed to assess the feasibility of implementing a PE programme (‘ACTIVA-Murcia’ Programme) recommended in the primary healthcare setting for primary prevention of ischaemic cardiovascular disease.

### Ethics

The study protocol was approved by the Ethics Committee for Clinical Research (ECCR act 10/10, November 29, 2010) of Hospital Virgen de la Arrixaca, Murcia, Spain. All participants gave written informed consent.

### Study population

Men and women aged 18–70 years presenting with sedentary behaviour and hypertension or dyslipidaemia were eligible. Sedentary behaviour defined as having an energy expenditure of ≤1.5 metabolic equivalents (METs) (e.g. equivalent to sitting or lying down) was quantified using the short version of the International Physical Activity Questionnaire (IPAQ) [18]. Treatment with at least one antihypertensive agent or one lipid-lowering agent for the previous 12 months was required for the diagnosis of hypertension or dyslipidaemia. Exclusion criteria were severe or terminal illness, diagnosis of ischaemic heart disease and/or cerebrovascular disease, severe mental disorder, presence of a chronic disease in which a programme of PE was contraindicated, and pregnant or breast-feeding women. Based on a criterion established by the American College of Sports Medicine (ACSM) of the need of an exercise testing prior to perform PE in diabetic subjects with duration of disease >10 years, diabetic participants were not eligible for inclusion in the study. Participants who met ACSM criteria for exercise test termination were also excluded. Data was collected for five years (2013–2017).

### Measurements, variables, observations

*Description of the intervention***.** The ‘ACTIVA-Murcia’ programme enhances actions from the primary care setting through the participation of 54 community health centres to stimulate healthy behaviours and lifestyles of the population through PE. Key features of the programme include: a) government supported, b) free participation, c) the link of primary health care professionals (general practitioner and nurse), d) access restricted to patients in whom the PE had been recommended by primary care physicians or nurses, e) exercises tailored to individual characteristics of patients, and f) a sport facility close to the patient’s home. Briefly, the programme is based on standardised physical exercises, organised in training circuits, which include potentiating exercises to improve aerobic fitness, strength, flexibility and balance, with adequate progression of training and adapted to the characteristics of the patients considering their limitations and risk factors.

Physicians and nurses of primary care centres throughout the Region of Murcia were specially trained in the characteristics of PE and the referral protocol for the programme. Also, they received regularly leaflets with information on the importance of PE prescription. Patients who were considered eligible by the primary care professionals were invited to participate in the programme and for those who accepted and signed the written informed consent, an electronic recommendation form of PE was sent by fax to the corresponding city council. Each city council contacted patients by telephone to start physical activities in a municipal sports centre close to the patient’s home.

The PE programme was developed over 10 weeks, three days a week, with a total of 30 sessions of 1 h duration each. Instructors were graduates in Sciences of Physical Activities and Sports, who were selected according to criteria of experience in similar programmes and specific training of PE in adults. They were responsible for teaching the exercises, and considered the physical condition of each participant to individualise the PE programme. The modified Borg dyspnoea scale [19] was used as a reference for the prescribed target intensity of exercise. As physical condition is related to age, groups formed by instructors were also set up according to age. The fact that it was a group training (`peers’) not an individual programme is also a key feature of the intervention. Data of each participant included in the programme were entered in an anonymised database.

### Study variables

The percentage of participants being recruited by primary care physicians or nurses who presented to sports centres to participate in the PE programme was measured and the percentage of participants who completed more than 20 sessions out of a total of 30 sessions was recorded.

Study variables were recorded at the beginning and end of the programme. Aerobic fitness was tested with the 2 km (1.4 miles) walking test and results are expressed as the estimated maximal oxygen uptake (VO2 max) in ml/kg/min. Muscle strength was evaluated with the overhead forward medicine ball throw test [20]. Flexibility was measured using the sit and reach/drawer test. Trunk flexion from sitting is a validated test to measure the flexibility of the hamstring complex and is included in the Eurofit test battery. Motor fitness was evaluated by a single balance test (Flamingo balance test), the score of which is the total number of attempts (falls or loss of balance) needed to accumulate a total stable balance time of 60 s while standing on one foot. Details of some of these exercises based on the Eurofit test battery for adults have been previously reported [21]. Anthropometric variables (weight, height, body mass index [BMI]) were also measured. Health-related quality of life was assessed using the Short Form (36) Health Survey (SF-36) (the higher the score, the less disability) [22].

At the end of the PE programme, patients completed a questionnaire regarding improvement in physical fitness and mood (categorised as ‘much better,’ ‘somewhat better,’ ‘about the same,’ ‘a little’), benefits provided by PE (‘flexibility and agility,’ ‘knowledge and motivation for physical exercising,’ ‘reduction of blood pressure or serum cholesterol levels,’ ‘improvement in well-being,’ ‘weight loss or reduction of body volume,’ ‘interpersonal relationships’) and willingness to continue exercising regularly in the next six months (‘yes, for sure,’ probably yes,’ ‘no, for sure,’ ‘probably no’).

### Data analysis

Results are expressed as frequencies and percentages for categorical variables and as mean and standard deviation (SD) for continuous variables. Differences in the study variables between baseline values and results at 10 weeks were assessed with the chi-square (Χ^2^) test for categorical variables and ANOVA for repeated measures with two study factors: within-subject factor (time: initial and final) and between-subject factor (sex: men and women) for paired data. The Bonferroni’s method was used for pairwise comparison. Statistical analysis was performed with the SPSS version 21.0 (IBM Corp., Armonk, NY, USA). Statistical significance was set at *p* < 0.05.

## Results

Of a total of 6,140 subjects to whom the PE programme was offered, 5,077 (82.7%) accepted to participate and received a recommendation of PE. Main reasons for refusal were difficulties of accessing the sports centre (e.g. lack of public transport facilities) and inconveniences with the time schedule. Of the 5,077 recruited subjects who gave consent and presented to sports centres to participate in the PE programme, 3,656 (72%) finally started the programme. A total of 2,962 patients (81% of the subjects who went to the sports centre and 58.3% of the recruited subjects) completed between 20 and 30 sessions, 523 (14.3%) between 10 and 20 sessions, and 171 (4.7%) at least 10 sessions.

The 3,656 patients (69.6% women) who started the programme consisted of 131 groups of 25–27 people. The mean age was 53.5 years (range 18–79 years). General practitioners accounted for 77.9% of patients’ referrals and nurses for the remaining 22.1%. Men have attended a mean (SD) percentage of PE sessions of 66.6 (34.9) and women a mean percentage of 71.4 (27.3) sessions (95% CI for the difference −7.19 to −2.43, *p* < 0.001).

As shown in Table 1, there was a statistically significant change in all study variables after 10 weeks of PE as compared with baseline, with decreases in body weight and BMI and improvements in aerobic fitness, muscle strength, flexibility and balance. Improvements were also seen in both men and women, although between-group differences (men vs. women) were only statistically significant for strength. Quality of life was also significantly better in all domains after completion of the programme (Table 2). Within-group differences were statistically significant in all domains in both men and women, except for ‘bodily pain’ in men. Between-group differences were only significant for ‘bodily pain’.

**Table 1.** Mean and 95% confidence interval of anthropometric variables and physical condition of patients who started the PE programme and those who finished the programme after 10 weeks.

Study variables	All participants			Men		Women		ANOVA for repeated measures with 2 study factors: time and sex Bonferronís method
	Baseline	After 10 weeks	*95% IC Differ. p*	Baseline	After 10 weeks	Baseline	After 10 weeks	
Body weight, kg	79.0 (78.2, 79.8)	78.5 (77.8, 79.2)	(−0.64, −0.19) *p* < 0.001	87.2 (85.9, 88.5)	87.1 (85.8, 88.4)	76.3 (75.5, 77.1)	75.6** (74.9, 76.3)	*p* = 0.025 F = 5.1
Body mass index, BMI, kg/m^2^	30.2 (29.9, 30.5)	29.9 (29.6, 30.2)	(−0.34, −0.20) *p* < 0.001	31.1 (30.7, 31.6)	30.8* (30.4, 31.2)	29.8 (29.6, 30.0)	29.6** (29.3, 29.9)	*p* = 0.393 F = 0.7
Physical condition								
Aerobic fitness, ml/min/kg	29.7 (29.2, 30.2)	32.2 (29.7, 32.7)	(2.32, 2.79) *p* < 0.001	32.0 (31.1, 32.9)	34.6** (33.7, 35.5)	28.9 (28.4, 29.4)	31.4** (30.8, 32.0)	*p* = 0.524 F = 0.4
Strength, m	5.4 (5.3, 5.5)	5.9 (5.8, 6.0)	(0.57, 0.67) *p* < 0.001	6.5 (6.3, 6.7)	7.3** (7.2, 7.4)	4.9 (4.8, 5.0)	5.3** (5.2, 5.4)	*p* < 0.001 F = 76.8
Flexibility, cm	1.6 (1.1, 2.1)	3.8 (3.4, 4.2)	(2.06, 2.63) *p* < 0.001	−0.52 (−1.3, 0.3)	2.1** (1.3, 2.9)	2.4 (1.8, 3.0)	4.8** (4.3, 5.3)	*p* = 0.057 F = 3.6
Balance, no. falls	2.1 (1.8, 2.3)	1.6 (1.5, 1.7)	(−0.60, −0.33) *p* < 0.001	2.1 (1.7, 2.4)	1.6** (1.3, 1.9)	2.1 (1.9, 2.3)	1.6** (1.5, 1.7)	*p* = 0.916 F = 0.01
	*N* = 3656	*N* = 2962		*N* = 1111	*N* = 900	*N* = 2545	*N* = 2062	

This analysis is performed for the total population and stratified by sex, and a comparison has been made by time (initial vs. final) and by sex x time.

* Statistically significant differences (*p* < 0.05) when compared to baseline.

** Statistically significant differences (*p* < 0.001) when compared to baseline.

95% IC Differ: 95% confidence interval for the difference.

F: F-Snedecor.

**Table 2.** Mean and 95% confidence interval of health-related quality of life of patients who started the PE programme and those who finished the programme after 10 weeks.

Study variables health-related quality of life	All participants			Men		Women		ANOVA for repeated measures with 2 study factors: time and sex Bonferronís method
	Baseline	After 10 weeks	*95% IC Differ. p*	Baseline	After 10 weeks	Baseline	After 10 weeks	
Physical functioning	75.3 (74.0, 76.6)	77.4 (76.2, 78.6)	(1.22, 3.03) *p* < 0.001	80.0 (77.8, 82.2)	82.2* (80.0, 84.4)	74.0 (72.9, 75.1)	76.1** (75.0, 77.2)	*p* = 0.870 F = 0.02
Role-physical	85.6 (83.8, 87.4)	90.1 (88.8, 91.6)	(2.33, 6.03) *p* < 0.001	88.6 (85.5, 91.7)	92.3* (89.7, 94.9)	84.7 (83.0, 86.4)	89.4** (87.9, 90.9)	*p* = 0.596 F = 0.3
Bodily pain	64.6 (63.1, 66.1)	68.4 (67.0, 69.8)	(1.56, 4.12) *p* < 0.001	70.5 (67.8, 73.2)	71.6 (69.1, 74.1)	63.0 (61.6, 64.4)	67.5** (66.2, 68.8)	*p* < 0.010 F = 6.6
General health	56.4 (55.2, 57.6)	58.9 (57.7, 60.1)	(1.70, 3.41) *p* < 0.001	60.2 (58.1, 62.3)	62.9** (60.9, 64.9)	55.4 (54.3, 56.5)	57.8** (56.8, 59.8)	*p* = 0.744 F = 0.1
Energy/fatigue (vitality)	56.6 (55.2, 58)	62.0 (60.7, 63.3)	(3.78, 5.82) *p* < 0.001	62.4 (59.9, 64.9)	66.3** (64.0, 68.6)	55.1 (53.8, 56.4)	60.8** (59.6, 62.0)	*p* = 0.088 F =2.9
Social functioning	73.7 (72.2, 75.2)	77.0 (75.6, 78.4)	(1.91, 4.36) *p* < 0.001	78.0 (75.4, 80.6)	80.8* (78.3, 83.3)	72.5 (71.2, 73.8)	76.0** (74.7, 77.3)	*p* = 0.593 F = 0.3
Role-emotional	89.0 (87.4, 90.6)	92.5 (91.2, 93.8)	(2.04, 5.04) *p* < 0.001	92.7 (90.0, 95.4)	96.2** (94.0, 98.4)	87.8 (86.3, 89.3)	91.4** (90.1, 92.7)	*p* = 0.969 F = 0.001
Mental health	64.0 (62.7, 65.3)	68.8 (67.6, 70.0)	(3.56, 5.57) *p* < 0.001	70.2 (67.9, 72.5)	74.3** (72.1, 76.5)	62.3 (61.2, 63.4)	67.4** (66.3, 68.5)	*p* = 0.351 F = 0.9
Change in health	54.4 (53.0, 55.8)	45.8 (44.3, 47.3)	(−10.1, −6.9) *p* < 0.001	52.4 (49.9, 54.9)	44.3** (41.6, 47.0)	54.9 (53.7, 56.1)	46.1** (44.8, 47.4)	*p* = 0.727 F = 0.1
	*N* = 3656	*N* = 2345		*N* = 2545	*N* = 1639	*N* = 1111	*N* = 706	

This analysis is performed for the total population and stratified by sex, and a comparison has been made by time (initial vs. final) and by sex x time.

* Statistically significant differences (*p* < 0.05) when compared to baseline.

** Statistically significant differences (*p* < 0.001) when compared to baseline.

95% IC Differ.: 95% confidence interval for the difference.

F: F-Snedecor.

OR: Odds ratio.

CI: Confidence interval.

**Table 3.** Number and percentage of subjects who gave a positive opinion to each of the items of the variable ‘benefits provided by the program’ and number and percentage of subjects who showed their willingness to continue exercising at 6 months.

	All patients *N* (%)	Men *N* (%)	Women *N* (%)	Benefits x sex *P*	OR (CI 95%)
Benefits provided by the programme					
Flexibility and agility	719 (43.9)	170 (44.1)	556 (44.4)	*p* < 0.001 (X^2^ = 27.8)	0.99 (0.78–1.25)
Knowledge and motivation for physical exercise	78 (4.8)	25 (6.4)	54 (4.3)		1.52 (0.92–2.52)
Reduction of blood pressure	32 (2.0)	9 (2.2)	23 (1.8)		1.22 (0.54–2.75)
Improvement in well-being	472 (28.9)	83 (21.5)	382 (30.5)		0.62 (0.47–0.82)*
Weight loss or reduction of body volume	80 (4.9)	33 (8.7)	48 (3.8)		1.72 (1.07–2.74)*
Interpersonal relationships	109 (6.7)	21 (5.6)	85 (6.8)		0.81 (0.49–1.34)
Other	146 (8.9)	44 (11.4)	103 (8.2)		1.44 (0.98–2.11)
Total patients	1636 (100)	385 (100)	1251 (100)		
Willingness to continue exercising regularly in the next 6 months					
Yes, for sure	1,068 (49.4)	277 (52.1)	792 (48.6)	*p* = 0.154 (X^2^ = 5.26)	
Probably yes	988 (45.7)	228 (43.0)	760 (46.6)		
No, for sure	11 (0.5)	0	10 (0.6)		
Probably no	94 (4.4)	27 (4.8)	68 (4.2)		
Total patients	2162 (100)	532 (100)	1630 (100)		

This analysis was performed of the patients who completed the satisfaction survey at the end of the exercise programme considered globally and stratified by sex, and a comparison by sex was made.

* Statistical significance (*p* < 0.05).

OR: Odds ratio.

CI: Confidence interval.

**Figure 1.**
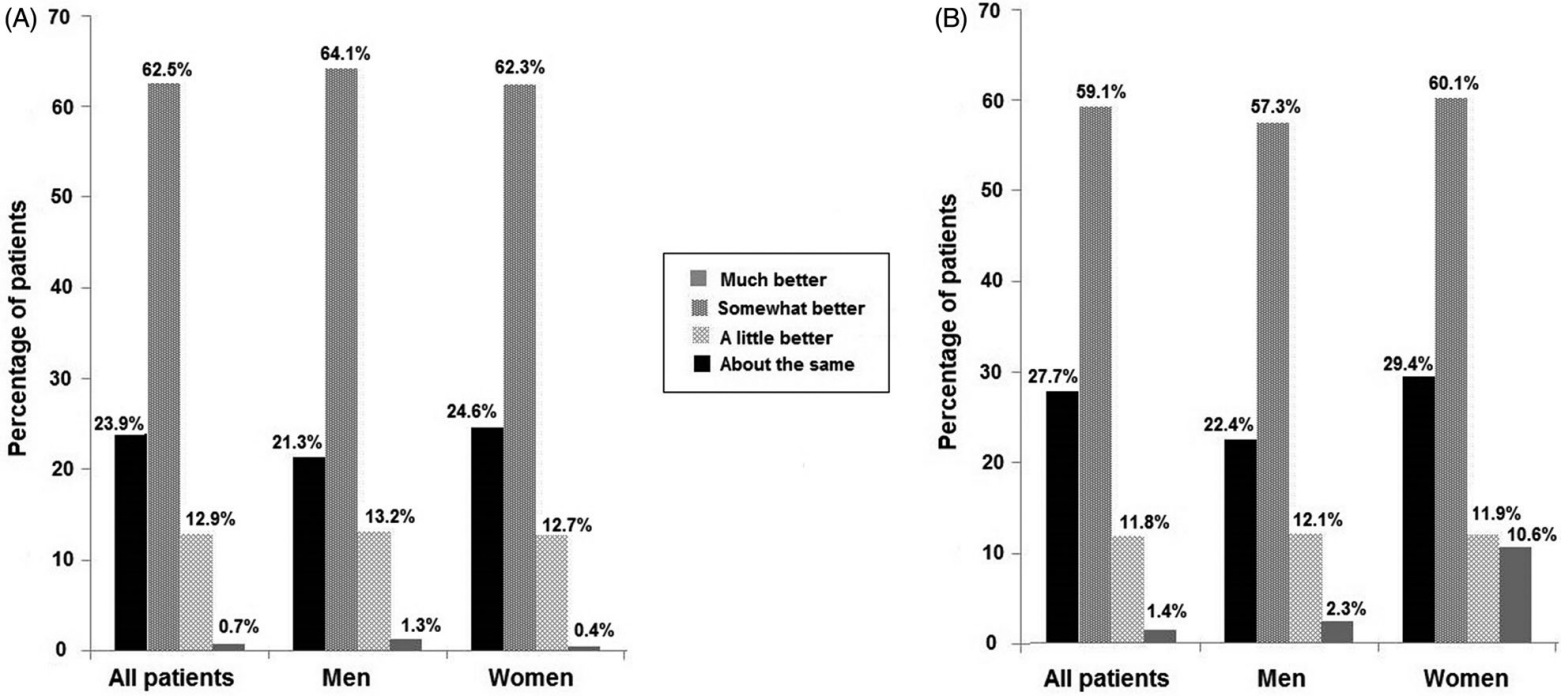
(A) Changes in physical fitness in men and women at 10 weeks as compared with baseline. (B) Changes in mood in men and women at 10 weeks as compared with baseline.

As shown in Figure 1(A), physical fitness was considered that has been improved (‘much better/somewhat better’) by a large majority of patients (86.4%), although differences by gender were not observed (*p* = 0.69). Similar results were obtained for improvement in mood (‘much better/somewhat’ better) in 86.7% of the patients, although in this case, there were statistically significant differences between men and women, with a higher percentage of women who rated their mood ‘about the same’ as compared with men (10.6% vs. 2.3%; *p* = 0.005) (Figure 1(B)).

About benefits provided by PE, ‘flexibility and agility’ and ‘improvement in well-being’ were rated as improved by 43.9% and 28.9%, respectively (Table 3). The comparison between men and women was statistically significant, with a higher percentage of women reporting improvements in well-being and weight loss, and a higher percentage of men reporting improvement of interpersonal relationships. Also, 49.4% of patients strongly believed that they would continue exercising regularly in the next 6 months and 45.7% considered this as probable (Table 3).

## Discussion

### Main findings

Participation in a 10-week PE programme for patients with sedentary behaviour and hypertension or dyslipidaemia recruited by general practitioners and nurses in the primary care setting was associated with decreases in body weight and BMI and improvements in physical condition and health-related quality of life. Improvements were seen in both men and women.

### Strengths and limitations

The strengths of the study include the high participation rate, the recommendation of PE by general practitioners and nurses, the fact that the programme was offered for free, and the facility to practice physical activities in a sports centre closest to the patient’s home. Limitations include that this study was not a randomised controlled trial, the short-term assessment of results, the lack of stratification by age groups and difficulties in the generalizability of results due to organisational differences of the healthcare systems. Also, positive results could have been influenced by the fact that teachers and outcome collectors were the same persons (not blinded). Benefits in patients with other medical conditions (e.g. diabetes) were not evaluated in this study.

### Interpretation

Health benefits, mainly improvement in physical fitness, obtained in this pre-post comparative study after the implementation of a tailored PE programme at the community level in patients with hypertension or dyslipidaemia are challenging to compare with other studies. This was not a randomised controlled trial and the focus of our study was the primary prevention of ischaemic heart disease in cardiovascular disease (CVD) risk patients. Community-based risk reduction interventions through single or integrated comprehensive multimodal strategies based on education programmes, medical screenings, improved knowledge and awareness, risk behaviour modification (lifestyle changes, smoking cessation, weight reduction, blood pressure control, etc.), behaviour change techniques or motivational approaches have been the objectives of numerous primary studies for DVD prevention and control. However, these studies differ in their settings, methods, components and intensity of the interventions, risk factors targets, evaluation methods, periods, design and duration of effects.

As far as we are aware, other primary care studies with components of our PE programme (free, in community close to patients, recommendation by general practitioners/nurses, tailored, group sessions with peers) have not been published. In Catalonia, one of the 17 autonomous communities in Spain, the local government developed its physical activity, sports and health plan to increase the proportion of adults complying with physical activity (PA) recommendations [23]. In this model, PA screening and advice in primary health care settings was based on stage of change, in which unprepared adults at the precontemplative or contemplative stage receive the motivational approach, inactive adults in the preparation stage receive specific advice with follow-up and information on accessible resources and activities for PA, and those in the active or maintenance stage receive reinforcement to prevent relapse. Moreover, motivational interview, the trans-theoretical stages of changes and individualised prescription of PE in routine primary care settings have been proposed as interventions in protocols of trials for patients with cardiovascular risk factors [24].

### Implications

Improvement in physical fitness and quality of life associated with a community-based PE programme should encourage general practitioners to prescribe PE to cardiovascular risk patients as a highly relevant primary preventive measure in their routine daily practice. An interesting finding is that people feel much better and many of them express the desire to continue exercising. The ‘ACTIVA-Murcia’ is an ongoing programme, with a current participation (2020) of 60 community health centres. We expect to collect and analyse data at 5-years intervals. However, affordability of the programme should be evaluated if this comes at a reasonable cost. The cost for each group of people is €750, with a total of €92,250 for the 131 groups. These are the expenses generated by the activity. Indirect expenses (programme management by the Health Council of the Region of Murcia) are €110,000. Also, about €120,000 annual costs of human resources should be added. In fact, we are currently developing a study with qualitative methodology whose objective is to analyse the factors that influence the adherence or non-adherence of the subjects to PE after the development of the programme.

## Conclusion

Implementation of a community-based PE programme at a regional level for cardiovascular risk patients from the general population is feasible. Important characteristics of the programme include the recommendation of PE by healthcare professionals in the primary care setting, offered for free to people with sedentary behaviour and organised by the local municipality.
